# Modeling Chemical Interaction Profiles: I. Spectral Data-Activity Relationship and Structure-Activity Relationship Models for Inhibitors and Non-inhibitors of Cytochrome P450 CYP3A4 and CYP2D6 Isozymes

**DOI:** 10.3390/molecules17033383

**Published:** 2012-03-15

**Authors:** Brooks McPhail, Yunfeng Tie, Huixiao Hong, Bruce A. Pearce, Laura K. Schnackenberg, Weigong Ge, James C. Fuscoe, Weida Tong, Dan A. Buzatu, Jon G. Wilkes, Bruce A. Fowler, Eugene Demchuk, Richard D. Beger

**Affiliations:** 1 Division of Toxicology and Environmental Medicine, Agency for Toxic Substances and Disease Registry, Atlanta, GA 30333, USA; Email: BMcPhail@cdc.gov (B.M.); YTie@cdc.gov (Y.T.); BFowler@icfi.com (B.A.F.); 2 Division of Systems Biology, National Center for Toxicological Research, U.S. Food and Drug Administration, Jefferson, AR 72079, USA; Email: huixiao.hong@fda.hhs.gov (H.H.); BruceA.Pearce@fda.hhs.gov (B.A.P.); Laura.Schnackenberg@fda.hhs.gov (L.K.S.); Weigong.Ge@fda.hhs.gov (W.G.); James.Fuscoe@fda.hhs.gov (J.C.F.); Weida.Tong@fda.hhs.gov (W.T.); Dan.Buzatu@fda.hhs.gov (D.A.B.); Jon.Wilkes@fda.hhs.gov (J.G.W.); Richard.Beger@fda.hhs.gov (R.D.B.); 3 Science and Research Staff, Office of Pharmaceutical Science, Center for Drug Evaluation and Research, U.S. Food and Drug Administration, Silver Spring, MD 20993, USA; Email: Luis.Valerio@fda.hhs.gov; 4 Department of Basic Pharmaceutical Sciences, West Virginia University, Morgantown, WV 26506-9530, USA

**Keywords:** structure-activity relationship, SAR, SDAR, classifier, cytochrome P450, inhibitor, CYP3A4, CYP2D6

## Abstract

An interagency collaboration was established to model chemical interactions that may cause adverse health effects when an exposure to a mixture of chemicals occurs. Many of these chemicals—drugs, pesticides, and environmental pollutant—interact at the level of metabolic biotransformations mediated by cytochrome P450 (CYP) enzymes. In the present work, spectral data-activity relationship (SDAR) and structure-activity relationship (SAR) approaches were used to develop machine-learning classifiers of inhibitors and non-inhibitors of the CYP3A4 and CYP2D6 isozymes. The models were built upon 602 reference pharmaceutical compounds whose interactions have been deduced from clinical data, and 100 additional chemicals that were used to evaluate model performance in an external validation (EV) test. SDAR is an innovative modeling approach that relies on discriminant analysis applied to binned nuclear magnetic resonance (NMR) spectral descriptors. In the present work, both 1D ^13^C and 1D ^15^N-NMR spectra were used together in a novel implementation of the SDAR technique. It was found that increasing the binning size of 1D ^13^C-NMR and ^15^N-NMR spectra caused an increase in the tenfold cross-validation (CV) performance in terms of both the rate of correct classification and sensitivity. The results of SDAR modeling were verified using SAR. For SAR modeling, a decision forest approach involving from 6 to 17 Mold^2^ descriptors in a tree was used. Average rates of correct classification of SDAR and SAR models in a hundred CV tests were 60% and 61% for CYP3A4, and 62% and 70% for CYP2D6, respectively. The rates of correct classification of SDAR and SAR models in the EV test were 73% and 86% for CYP3A4, and 76% and 90% for CYP2D6, respectively. Thus, both SDAR and SAR methods demonstrated a comparable performance in modeling a large set of structurally diverse data. Based on unique NMR structural descriptors, the new SDAR modeling method complements the existing SAR techniques, providing an independent estimator that can increase confidence in a structure-activity assessment. When modeling was applied to hazardous environmental chemicals, it was found that up to 20% of them may be substrates and up to 10% of them may be inhibitors of the CYP3A4 and CYP2D6 isoforms. The developed models provide a rare opportunity for the environmental health branch of the public health service to extrapolate to hazardous chemicals directly from human clinical data. Therefore, the pharmacological and environmental health branches are both expected to benefit from these reported models.

## 1. Introduction

Interactions between multiple drugs and between drugs and other chemical compounds may be responsible for many human adverse health effects; however, such effects are not yet fully understood or routinely studied. Various combinations of drug therapies, lifestyle choices (e.g., consumption of alcohol or nutritional supplements) and environmental factors (e.g., contaminated drinking-water and air at hazardous waste sites [HWS]), may increase occurrences of the drug-drug/chemical interactions (DDCIs). Drugs and environmental chemicals are often eliminated from the body by means of similar phase I and phase II biotransformations, and some of the compounds can either induce or inhibit enzymes involved in these processes. Cytochrome P450 (CYP) enzymes metabolize a wide range of xenobiotics (pharmaceutical and environmental compounds) via the phase I metabolism. Usually, this involves the oxidation, reduction, hydrolysis, dehydrogenation/hydrogenation, or mono-oxygenation of compounds.

CYPs comprise a large class of isozymes, each catalyzing the biotransformation of similar compounds. CYP1A2, CYP2B6, CYP2C9, CYP2C19, CYP2D6, and CYP3A4 are the major CYP isozymes, responsible for the metabolism of drugs in the human liver [1]. CYP3A4 constitutes approximately 40% of the total CYP isozymes present in the adult liver, and it metabolizes more than 50% of xenobiotics that enter the body [2,3]. Unlike other CYPs, CYP3A4 has the greatest range of substrate specificity, and is capable of metabolizing compounds of different chemical classes that vary in molecular weight and shape [3,4]. While CYP2D6 constitutes only 2% of the total hepatic CYP, it is involved in metabolism of as much as 30% of known drugs [2,5]. CYP2D6 substrates are generally lipophilic bases with an aromatic ring and nitrogen atom [6]. The co-addition of inhibitors or substrates of CYP2D6 and CYP3A4 may alter their ability to biotransform other drugs. Because of the large number of xenobiotics, whose clearance depends on CYP3A4 and CYP2D6, inhibition of these isozymes has the greatest potential to cause DDCIs.

The presence of xenobiotics in the body can cause alterations of drug metabolism. For example, enzymatic inhibitors cause substrates (e.g., drugs) to compete for enzymes and may alter the enzymatic rate and extent of metabolism. Competitive inhibitors generally resemble the substrates and disrupt binding of drugs and chemicals by ligating to the substrate-binding site on the enzyme. Competitive inhibitors decrease the number of active enzyme sites, resulting in an increased parent-drug concentration in blood and tissues. Noncompetitive inhibitors do not necessarily resemble the substrate but still produce an inhibitory effect by binding to a site other than the catalytic site of the enzyme, causing allosteric transformations that reduce the rate of the enzymatic reactions. Any chemical that is metabolized by CYP enzymes may potentially alter the metabolism of other drugs, chemicals or both that are also substrates of these enzymes. An altered blood concentration of the drug may increase the potential for adverse health effects [7,8,9,10,11,12,13].

Information about DDCIs that can occur in patients is presented on drug labels [14] and at the U.S. Food and Drug Administration (FDA) drug interaction Web site [15]. Usually, minimal data are available on how a new clinical drug will interact with other drugs and chemicals. Often DDCIs are comprehensively described only after drugs have been prescribed to patients and used on the market for a prolonged period of time. DDCIs may modify the desired therapeutic outcome in patients and potentially cause adverse health effects.

Structure-activity relationship (SAR) modeling is used in drug discovery and for toxicological risk assessment of compounds [16,17,18,19]. The use of ^13^C nuclear magnetic resonance (NMR) chemical shifts as SAR descriptors has similar applications [16]; this has been referred to as spectral data-activity relationship (SDAR) modeling [20]. SDAR is a hybrid between a typical SAR approach, which relates substructural fragments or molecular properties of the compound to the biological or toxicological activity of the compound, and a 3D-QSAR (three-dimensional quantitative SAR) approach that relates physical fields around the chemical to its activity [21].

NMR spectroscopy is a major physical-chemical technique used for interrogating the atomic spatial organization of a compound. It can be very sensitive to structural re-arrangements at the atomic level and, as such, is widely used for solving 3D-structures of molecules. NMR can provide insights into secondary and tertiary structures, molecular dynamics, and mechanisms and products of chemical reactions. NMR reports information about local physical–chemical environment of a query atom and, in this sense, mimics a molecular field and orbital configuration around the nucleus.

Previously, the SDAR methodology has been employed only in the development of models based upon the ^13^C-NMR chemical shifts [20]. That implementation works well for carbon-rich organic chemicals [22,23,24], although it may be insufficient for adequate description of nitrogenated compounds. Lipophilic bases with a basic nitrogen atom are the major substrates of CYP2D6; the substrate nitrogen atom binds to aspartic acid on CYP2D, and substrate oxidation takes place at a distance of 5–7 Å from the nitrogen atom [25]. In the present work, we developed a combined ^13^C and ^15^N-NMR SDAR approach, which is expected to better describe the binding properties of xenobiotics specific to CYP enzymes.

SDAR/SAR/QSAR modeling relies on “supervised machine learning” techniques. Its success depends on the quality and quantity of data that are used in the learning process. Recently, it has become increasingly popular to rely on large high-throughput screening (HTS) libraries of *in vitro* data for DDCI model development [26,27,28,29,30]. Our own investigation [31] and multiple literature sources [32,33,34,35,36] suggest exercising a conservative approach when interpreting and using *in vitro* information for making decisions about clinical DDCIs. A complete understanding of *in vitro* to *in vivo* extrapolation is still emerging [37]. Accordingly, the current practice of inscribing drug labels is based on pharmaco-kinetic (PK) data from clinical studies, while using *in vitro* information is recommended in drug discovery and preclinical assessment of DDCI liabilities [38]. The PK data represent a cumulative characteristic of the whole-body response, not just inhibition at the CYP/CYP-reductase level, which is expressed by standard *in vitro* assays. Confusion about practical relevance of *in vitro* data and a high degree of false positives as compared with PK DDCIs results in clinicians overriding approximately 90% of DDCI alerts [39]. Also, a typical *in vitro* bioassay library consists predominantly of “drug candidates”, most, if not all, of which will never become a drug. Since these compounds have not been approved by FDA, their clinical relevance is questionable (as well as the relevance of a chemical space, which they represent, to the chemical space of actual FDA-approved drugs). Our own analysis of PubChem libraries that are available for CYP3A4 and CYP2D6 isozymes [40] suggests only a small overlap between chemicals in the libraries and clinical drugs on the market (see the Experimental section that follows). Since the ultimate goal of a machine classifier is to prevent actual DDCIs in the population, it is desirable to choose a learning domain of the model in the chemical space as close as possible to pharmaceuticals on the market. Furthermore, *in vitro* HTS data that lack statistical power shall not be used for model development. Because of the aforementioned reasons, in the present work, curated data from a well-known dataset [41] were employed for supervised learning.

Interpretation of *in vitro* data for CYP3A4 inhibition is especially challenging [32,33,34,35,36,42] because of atypical kinetics and multiple binding sites on the enzyme [43,44,45,46]. To address the challenge of indiscriminate ligand binding, a multiple pharmacophore hypothesis has been proposed for modeling CYP3A4 HTS data, which implies a SAR machine classifier as an adjunct [27]. In that work, the authors have implemented a support vector machine (SVM) classifier that is 95% and 75% accurate with respect to the training and 5-fold cross-validation sets. This example demonstrates that uniformity of data in the training set, which at first may be thought of as an advantage of a “uniform” simplified enzyme system in HTS screening, and which used to be a prerequisite for traditional QSARs, is no longer an obligation with modern model-building approaches, of course if the minority populations are statistically adequately represented by the training set. In fact, machine learning has been specifically developed to deal with heterogeneous data. Similarly to the aforementioned non-uniformity in the HTS data, modern modeling techniques have the potential to appropriately handle non-uniformity of multi-level inhibition processes embedded in clinical, toxicological data or both [18,19,47].

In the present work, multiple *in silico* SDAR and SAR classifier models were developed to estimate DDCIs that involve CYP3A4 and CYP2D6 isozymes. Inhibitors and non-inhibitors of the CYP3A4 and CYP2D6 isozymes were used to develop SDAR and SAR models. The presented DDCI modeling may help regulatory agencies to fulfill their missions and may contribute to improved public health. Accurate machine models could be used to identify perpetrators of CYP-mediated biotransformations, which, when complemented by routine *in vitro* and *in vivo* metabolism and transport studies, can help delineate CYP3A4- and CYP2D6-mediated DDCIs for new drugs and environmental pollutants. This will allow for assessment of risk of adverse events before they are observed and reported in the clinic [37,38].

## 2. Results and Discussion

### 2.1. SDAR Models of Inhibitors and non-Inhibitors of CYP3A4 and CYP2D6

Results of the tenfold (leave 10% out) cross-validation (CV) for the linear discriminant analysis (LDA) classification SDAR (SDAR LDA) models of inhibitors and non-inhibitors of CYP3A4 are summarized in Table 1. These results were obtained using 1 ppm ^13^C and 5 ppm ^15^N (C1&N5) bins, 2 ppm ^13^C and 10 ppm ^15^N (C2&N10) bins, and 3 ppm ^13^C and 15 ppm ^15^N (C3&N15) bins. The specificity, sensitivity, and rate of correct classification of training models decreased as the size of the bins was increased from C1&N5 to C3&N15; however, during CV the sensitivity of models built using the forward stepwise discriminant function analysis with criteria for entry and removal of the terms set using the calculated values of Fisher’s *F*-distribution, *F* > 0.5 and *F* > 2.0, and the rate of correct classification of models with *F* > 2.0, increased as the size of the bins was increased from C1&N5 to C3&N15. Table 2 shows the number of selected bins over the number of available bins for the CYP3A4 SDAR LDA model. An increase in the number of selected bins was evident for *F* > 0.5 as compared with *F* > 2.0. Table 3 shows the sensitivity, specificity, and rate of correct classification of the inhibitor/non-inhibitor SDAR LDA models for CYP3A4 calculated using an external validation (EV) set of 100 compounds. The rate of correct classification of EV for the six CYP3A4 SDAR LDA models ranged from 67% to 73%. Of the three bin combinations, the C2&N10 models performed best in terms of rate of correct classification and specificity. However, in terms of sensitivity the C1&N5 | *F* > 0.5 and C3&N15 | *F* > 2.0 models outperformed. These results showed that the SDAR LDA models for CYP3A4 were fairly consistent in the rate of correct classification of their estimates across a range of bin sizes. Some of the significant bins for C3&N15 | *F* > 2.0 SDAR LDA models were: C3 (102–105 ppm), which appeared to detect aromatic ring carbons from many inhibitors that were dipines and antibiotics; C3 (96–99 ppm), which detected ester carbons from sugar moieties in inhibitors that were mycins; and N15 (330–345 ppm) and (225–240 ppm), which appeared to detect compounds with heterocyclic rings that contain two or more nitrogen atoms in the rings. This type of structural information showed how the SDAR models could be used in reverse to infer relationships between the structure and activity. Descriptors useful for modeling in the decision forest (DF) approach are not nearly as informative because usually they are not associated with a single structural feature.

**Table 1:** Results of training and tenfold cross-validation for SDAR LDA and SAR DF models of inhibitors and non-inhibitors of CYP3A4.

Model type	Data set	Forward stepwise analysis criterion	Correct classification *	Sensitivity *	Specificity *
**C1&N5**	Training	*F* > 0.5	83.4 ± 6.5	78.3 ± 8.4	86.3 ± 6.1
**C2&N10**	Training	*F* > 0.5	76.1 ± 0.6	68.3 ± 1.0	80.4 ± 0.6
**C3&N15**	Training	*F* > 0.5	72.5 ± 0.6	65.1 ± 1.0	76.7 ± 0.8
**C1&N5**	CV	*F* > 0.5	56.9 ± 12.3	42.7 ± 21.2	64.9 ± 16.7
**C2&N10**	CV	*F* > 0.5	57.2 ± 2.2	43.3 ± 5.9	64.9 ± 5.1
**C3&N15**	CV	*F* > 0.5	58.6 ± 2.9	45.9 ± 6.1	65.6 ± 3.5
**C1&N5**	Training	*F* > 2.0	80.0 ± 0.4	73.4 ± 0.8	83.7 ± 0.6
**C2&N10**	Training	*F* > 2.0	72.6 ± 0.6	60.9 ± 1.0	79.1 ± 0.6
**C3&N15**	Training	*F* > 2.0	70.1 ± 0.6	58.1 ± 1.0	76.9 ± 0.6
**C1&N5**	CV	*F* > 2.0	56.6 ± 2.5	41.9 ± 5.3	64.8 ± 4.3
**C2&N10**	CV	*F* > 2.0	58.8 ± 2.5	42.1 ± 3.5	68.1 ± 3.3
**C3&N15**	CV	*F* > 2.0	59.8 ± 2.5	44.2 ± 5.5	68.5 ± 2.2
**SAR**	CV	NA	61.0 ± 2.0	39.0 ± 3.9	74.0 ± 2.0

* Rates are in percent; 95% confidence intervals are shown.

**Table 2:** SDAR LDA model for CYP3A4 showing number of bins selected/number of bins available.

Forward stepwise analysis criterion	C1&N5	C2&N10	C3&N15
*F* > 0.5	99/273	70/147	49/99
*F* > 2.0	63/273	31/147	23/99

**Table 3:** External validation statistics (%) of the CYP3A4 models.

Model type	Forward stepwise analysis criterion	Correct classification	Sensitivity	Specificity
**C1&N5**	*F* > 0.5	70	60	73
**C2&N10**	*F* > 0.5	72	52	79
**C3&N15**	*F* > 0.5	70	56	75
**C1&N5**	*F* > 2.0	67	48	73
**C2&N10**	*F* > 2.0	73	52	80
**C3&N15**	*F* > 2.0	70	56	75
**SAR**	NA	86	76	89
**ACD/ADME^™^, “general” mode**	NA	68	56	72
**ACD/ADME^™^, “efficient” mode**	NA	68	12	87

Table 4 displays the tenfold CV results for SDAR models of inhibitors and non-inhibitors of CYP2D6 using C1&N5, C2&N10, and C3&N15 binning. Similar to results reported above, the specificity, sensitivity, and rate of correct classification of the training models decreased as the size of the bins was increased from C1&N5 to C3&N15. At the same time, the rate of correct classification and sensitivity of the CV classification increased as the size of the bins was increased from C1&N5 | *F* > 2.0 to C3&N15 | *F* > 2.0 SDAR LDA models. For C1&N5, 100 of total 272 bins for the SDAR LDA models with *F* > 0.5 and 63 of 272 bins with *F* > 2.0 were selected (Table 5). For C2&N10, 78 of 147 possible bins with *F* > 0.5 and 30 of 147 bins with *F* > 2.0 were selected. For C3&N15, 60 of 99 possible bins with *F* > 0.5 and 23 of 99 bins with *F* > 2.0 were selected. The top two bins for the C3&N15 SDAR model for CYP2D6 were: C3 (144–147 ppm), which selected carbons from many inhibitors that were dipines, and C3 (48–51 ppm), which selected carbon atoms that were from the classes off dipines and beta blockers (olols).

**Table 4:** Results of training and tenfold cross-validation for SDAR LDA and SAR DF models of inhibitors and non-inhibitors of CYP2D6.

Model type	Data set	Forward stepwise analysis criterion	Correct classification *	Sensitivity *	Specificity *
**C1&N5**	Training	*F* > 0.5	85.6 ± 2.0	86.0 ± 3.5	85.5 ± 2.2
**C2&N10**	Training	*F* > 0.5	79.0 ± 0.6	80.5 ± 1.2	78.4 ± 0.6
**C3&N15**	Training	*F* > 0.5	74.1 ± 0.4	75.1 ± 0.8	73.7 ± 0.4
**C1&N5**	CV	*F* > 0.5	61.6 ± 12.0	47.6 ± 25.5	66.8 ± 13.1
**C2&N10**	CV	*F* > 0.5	61.3 ± 3.1	50.3 ± 5.7	65.3 ± 2.7
**C3&N15**	CV	*F* > 0.5	60.5 ± 3.1	51.9 ± 4.3	65.6 ± 4.3
**C1&N5**	Training	*F* > 2.0	82.1 ± 0.6	80.9 ± 1.6	82.5 ± 0.6
**C2&N10**	Training	*F* > 2.0	74.4 ± 0.6	72.1 ± 0.8	75.2 ± 0.8
**C3&N15**	Training	*F* > 2.0	70.4 ± 0.6	68.7 ± 1.4	71.0 ± 0.6
**C1&N5**	CV	*F* > 2.0	60.2 ± 2.7	45.6 ± 5.3	65.4 ± 3.1
**C2&N10**	CV	*F* > 2.0	60.7 ± 3.3	50.3 ± 5.7	64.6 ± 3.9
**C3&N15**	CV	*F* > 2.0	61.8 ± 3.5	54.7 ± 6.7	64.5 ± 5.3
**SAR**	CV	NA	70.0 ± 3.9	26.0 ± 5.9	85.0 ± 5.9

* Rates are in percent; 95% confidence intervals are shown.

**Table 5:** SDAR LDA model for CYP2D6 showing number of bins selected/number of bins available.

Forward stepwise analysis criterion	C1&N5	C2&N10	C3&N15
*F* > 0.5	100/272	78/147	60/99
*F* > 2.0	63/272	30/147	23/99

Table 6 shows the sensitivity, specificity, and rate of correct classification of the inhibitor/non-inhibitor SDAR LDA models of CYP2D6 tested with the EV set of 100 chemical entities. The rate of correct external classification of the six SDAR LDA models for CYP2D6 ranged from 66% to 76%, with C2&N10 models performing best in terms of rate of correct classification and specificity.

**Table 6:** External validation statistics (%) of the CYP2D6 models.

Model type	Forward stepwise analysis criterion	Correct classification	Sensitivity	Specificity
**C1&N5**	*F* > 0.5	68	45	74
**C2&N10**	*F* > 0.5	71	60	74
**C3&N15**	*F* > 0.5	70	55	74
**C1&N5**	*F* > 2.0	66	45	71
**C2&N10**	*F* > 2.0	76	70	78
**C3&N15**	*F* > 2.0	68	60	70
**SAR**	NA	90	60	98
**ACD/ADME^™^, “general” mode**	NA	76	55	81
**ACD/ADME^™^, “efficient” mode**	NA	62	30	74

Comparison of Table 2 and Table 5 demonstrated that a similar number of bins was used in the SDAR LDA models for CYP3A4 and CYP2D6, respectively. A closer inspection of the SDAR LDA models for CYP3A4 and CYP2D6 with *F* > 2.0 showed that even though the same number of bins was used to develop the models, the actual bins used in the models for CYP3A4 and CYP2D6 had a small overlap. The C3&N15 | *F* > 2.0 SDAR LDA models for CYP3A4 and CYP2D6 were each based on 23 of 99 potential bins, but only three of the 23 bins C3 (129–132), C3 (144–147), and C3 (198–201) were used in both models. The mean bin occupancy value of non-inhibitors was lower than for inhibitors in C3 (129–132) and C3 (144–147) bins for both the CYP3A4 and CYP2D6 models; in the C3 (198–201) bin, the mean occupancy value of inhibitors was higher than for non-inhibitors in the CYP3A4 models, and lower than for non-inhibitors in CYP2D6 models. Table 7 shows the mean bin occupancies and associated *p*-values for several select bins used in SDAR LDA models with *F* > 2.0. There were spectral regions in the C3&N15 models that were used extensively for either CYP3A4 or CYP2D6. For example, C3&N15 models for CYP3A4 used five of six bins between 60 ppm and 78 ppm, and in this region the mean bin occupancy of inhibitors was higher than the mean bin occupancy of non-inhibitors. The C2&N10 | *F* > 2.0 SDAR LDA models for CYP3A4 and CYP2D6 were each based on 30 and 31 bins, respectively, of 147 potential bins. Only six of the selected bins-*i.e.*, of 30 or 31 –C2 (32–34), C2 (42–44), C2 (146–148), C2 (184–186), C2 (198–200), and C2 (200–202) were the same. Interestingly, the spectral range of C3 (144–147) and C2 (146–148) bins overlapped, and the spectral range of the C3 (198–201) bin overlapped with C2 (198–200) and C2 (200–202) bins. In addition, similar to the C3&N15 models, several bins between 60 ppm and 78 ppm were used only in the CYP3A4 models, namely the C2 (60–62), C2 (62–64), C2 (72–74), and C2 (74–76) bins. The C3&N15 SDAR model for CYP2D6 relied on C3 (45–48) and C3 (48–51) bins, and the C2&N10 SDAR model for CYP2D6 relied on C2 (46–48) and C2 (48–50) bins. The C1&N5 | *F* > 2.0 SDAR LDA models for CYP3A4 and CYP2D6 were each based on 63 of 273 or 272 potential bins, respectively; 18 of the 63 bins were the same, and many of these smaller bins overlapped with the spectral bins used in the C2&N10 and C3&N15 SDAR LDA models.

**Table 7:** Mean values and *p*-values of selected bins used in C1&N5, C2&N10, and C3&N15 SDAR LDA models with *F* > 2.0.

SDAR Model Bin (ppm range)	CYP3A4			CYP2D6		
	mean bin occupancy *		LDA model *p*-value	mean bin occupancy *		LDA model *p*-value
	non-inhibitor	inhibitor		non-inhibitor	inhibitor	
**C1&N5**						
C1 (45)	8.29	19.44	0.000084	9.73	18.75	0.0025
C1 (49)	NA	NA	NA	9.28	15.00	0.056
C1 (63)	3.37	8.80	0.15	NA	NA	NA
C1 (147)	9.84	15.74	0.008	8.14	23.75	0.0000004
C1 (198)	0.52	0.46	0.14	0.45	1.25	0.024
N5 (235–240)	NA	NA	NA	2.94	6.88	0.031
**C2&N10**						
C2 (45–46)	29.02	35.19	0.11	NA	NA	NA
C2 (49–50)	NA	NA	NA	21.04	32.50	0.0033
C2 (63–64)	6.48	16.20	0.051	NA	NA	NA
C2 (146–148)	19.17	29.17	0.044	17.19	36.88	0.00048
C2 (198–200)	1.55	4.17	0.024	2.71	0.62	0.00051
N10 (230–240)	NA	NA	NA	8.82	16.25	0.0014
**C3&N15**						
C3 (42–45)	31.87	54.63	0.00044	NA	NA	NA
C3 (48–51)	NA	NA	NA	32.13	47.50	0.00083
C3 (63–66)	9.84	23.61	0.022	NA	NA	NA
C3 (144–147)	27.46	38.89	0.14	25.79	48.75	0.00022
C3 (198–201)	2.59	4.63	0.065	3.85	0.63	0.0073

* Mean bin occupancy of 100 implies one chemical shift in the specified range for every compound. NA means bin was not used in the SDAR DF model.

### 2.2. SAR DF Models of Inhibitors and Non-Inhibitors of CYP3A4 and CYP2D6

As shown in the last row of Table 1, the mean tenfold CV rate of correct classification, sensitivity, and specificity of SAR DF models for CYP3A4 were 61%, 39%, and 74%, respectively. The SAR row of Table 3 shows the rate of correct classification (86%), sensitivity (76%), and specificity (89%) of the model applied to the EV set of CYP3A4. The top five descriptors that contributed to the CYP3A4 SAR model were: the principal quantum vertex connectivity order-3 index; topological structure autocorrelation length-1 weighted by atomic masses; lowest eigenvalue from Burden matrix weighted by atomic polarizabilities order-1; Moran topological structure autocorrelation length-6 weighted by atomic masses; and Geary topological structure autocorrelation length-1 weighted by atomic polarizabilities. All descriptors were associated with molecular connectivity and shape. At the same time, the physical-chemical properties, such as polarizability and mass, were also present. These properties were fundamentally connected to NMR. NMR measures chemical shifts, which originate from nuclear magnetic moments in an external magnetic field. Therefore, the field shielding effect at the nucleus depends on the electronic environment of the nucleus, electronic polarizability or both. The NMR spectra are related to electronic polarizabilities through electrostatic potential changes and associated changes to the atomic electronic orbitals. Selection of the electronic-wise properties by the SAR DF machine-learning algorithm among a large number of less informative descriptors [48] suggested an implicit association between SAR DF descriptors and SDAR LDA bins, which incorporate the NMR spectra.

The mean tenfold CV rate of correct classification, sensitivity, and specificity of SAR DF models of inhibitors and non-inhibitors of CYP2D6 were 70%, 26%, and 85%, respectively (Table 4). As shown in Table 6, the rate of correct classification, sensitivity, and specificity of SAR DF models of inhibitors and non-inhibitors calculated for the EV set of CYP2D6 were 90%, 60%, and 98%, respectively. The top five contributors to the inhibitor/non-inhibitor SAR DF model of CYP2D6 were the: the mean atomic electronegative Allred-Rochow scaled on carbon; hydrophilic factor index; Moran topological structure autocorrelation length-6 weighted by atomic polarizabilities; mean electrotopological states index; and mean atomic electronegativity Sanderson-scaled on carbon. Similar to the CYP3A4 model, the molecular connectivity and shape were important. However, there was a greater diversity in the chemical-physical properties of descriptors that entered the model. In addition to atomic polarizabilities, atomic electronegativity and hydrophilicity were also selected in the process of model training. Therefore, the physical-chemical palette of the SAR DF model for CYP2D6 appeared to be richer than the one for CYP3A4. The latter, perhaps, is associated with a greater substrate specificity of the CYP2D6 isozyme, which introduces more severe constraints on the distribution of physical-chemical properties across the binding molecules. Similar to the CYP3A4 model, the selected physical-chemical descriptors indicated an association between the major SAR DF model descriptors and the SDAR LDA NMR spectra, because each chemical shift in the NMR spectrum of a compound is related to electronegativity of nearby atoms, shielding the nucleus from the interrogating magnetic field.

### 2.3. Analysis and Comparison of the SDAR LDA and SAR DF Models

The training rate of correct classification, sensitivity, and specificity of SDAR LDA inhibitor/non-inhibitor classification models for both CYP3A4 and CYP2D6 datasets decreased with increasing bin size. This occurred regardless whether the SDAR LDA model was trained using *F* > 0.5 or *F* > 2.0. The *F* > 0.5 training models for CYP3A4 were about 2–4% more accurate, 5–8% more sensitive, and as much as 3% more specific than the *F* > 2.0 models, which could be understood from the information content perspective. When the spectral information was distributed across a larger number of (smaller) bins, over-fitting more likely was expected to occur. At the same time, the rate of correct classification, sensitivity, and specificity of tenfold CV for SDAR LDA classification for inhibitors and non-inhibitors of CYP3A4 increased as the bin size increased. The more populated the spectral bin was, the greater was the amount of binned information and, therefore, the more reproducible was the statistical weight it provided. Accordingly, the reduced number of more populated bins helped reduce overtraining. The tenfold CV models for CYP3A4 also suggested that optimum sizes for ^13^C and/or ^15^N-NMR bins have not yet been reached. Only 1 ppm, 2 ppm, and 3 ppm ^13^C-NMR bin widths and 5 ppm, 10 ppm, and 15 ppm ^15^N-NMR bin widths were evaluated. Whether the bin sizes of either or both ^13^C and ^15^N needed to be increased or just one of them to obtain the optimal rate of correct classification, sensitivity, and specificity during the tenfold CV was unclear. Based upon previous experience with ^13^C, the ^13^C bin sizes were believed to be near optimal; however, further work may be required to optimize the ^15^N bin sizes in which the population of nitrogen atoms in chemicals are much lower and the spectral range is much larger compared with ^13^C. Similar effects were observed for SDAR LDA inhibitor and non-inhibitor models of CYP2D6, in which the rate of correct classification, sensitivity, and specificity during the training stage decreased as bin sizes increased, but the sensitivity of the SDAR LDA models (for both *F* > 0.5 and *F* > 2.0) increased with the increasing bin size. The present study is the first in which the effects of ^13^C and ^15^N bin sizes on model development were investigated, although the optimal bin sizes, especially for ^15^N, still need to be determined.

Noteworthy, how the SDAR LDA bins for the same spectral regions were selected in all three C1&N5, C2&N10, and C3&N15 SDAR LDA models summarized in Table 7. In certain cases, a carbon chemical shift at around 49 ppm was used in all three SDAR LDA models for CYP2D6, and it was significant in all three models. Bins C1 (49), C2 (49–50), and C3 (48–51), all had mean occupancies of inhibitors that were about 50% higher than the mean bin occupancies of non-inhibitors. The chemical shift at 147 ppm was selected in SDAR DF models for both CYP3A4 and CPY2D6 isozymes at all bin sizes. The only model where the bin at 147 ppm did not have a significant *p*-value was the C2&N10 SDAR LDA model for CYP3A4. Chemical shifts at around 147 ppm were primarily detecting carbon atoms next to heterocyclic nitrogen atoms in aromatic rings in compounds like felodipine, lercanidipine, and atorvastin. The carbon chemical-shift bin near 198 ppm was selected in all SDAR LDA models for both CYP3A4 and CYP2D6 isozymes. The bin had a significant *p*-value in all SDAR LDA models for CYP2D6 but the *p*-value was only significant for the C2&N10 SDAR LDA model of CYP3A4. The mean bin occupancy for bins near 198 ppm of inhibitors was lower than non-inhibitors in the C2&N10 and C3&N15 SDAR LDA models of CYP2D6, and it was higher in the C2&N10 and C3&N15 models of CYP3A4.

The tenfold CV results of the SDAR LDA and SAR DF classification models for inhibitors and non-inhibitors of CYP3A4 and CYP2D6 had similar rates of correct classification. The C3&N15 SDAR LDA models tended to have higher sensitivity than SAR DF models (44% to 39% for CYP3A4 and 55% to 26% for CYP2D6), while SAR DF tended to have a little more accuracy than the SDAR LDA models (61% to 60% for CYP3A4 and 70% to 62% for CYP2D6). The higher rate of correct classification of SAR DF models was caused by higher specificity, and SAR DF models performed better than SDAR LDA models in EV using the set of 100 compounds. SAR DF had the rate of correct classification as high as 86% and 90% for CYP3A4 and CYP2D6, respectively. That was greater than the rate of correct classification of SAR DF model in tenfold CV by 15% and 30%, respectively. Much of this increase was related to increased sensitivity, which increased from 39% to 76% for CYP3A4 and from 26% to 60% for CYP2D6. The sensitivity of SAR DF modeling during tenfold CV was driven by the fraction of inhibitors in the training data set, which was 36% for CYP3A4 and 26% for CYP2D6.

The rate of correct classification calculated for SDAR LDA models using the EV set was 12–15% greater compared with the tenfold internal CV. The increase in the rate of correct classification of SDAR LDA inhibitor and non-inhibitor models was due to both an increase in sensitivity and specificity (7–18% increase in sensitivity and 6–14% increase in specificity). Yap and Chen [41] have built accurate SVM models using the training data but did not report CV. Therefore, in terms of CV, a direct comparison between their SVM models and the reported SAR DF and SDAR LDA models is not feasible.

In the present work, the SDAR LDA and SAR DF models did not perform as effectually as Yap and Chen models [41], but Yap and Chen used SVM with a very large number of parameters—somewhat between 100 and 1,000. The authors mention that for CYP3A4 and CYP2D6 inhibitor and non-inhibitor models the peak of model performance was reached at about 400 parameters, although the exact number has not been reported. This circumstance could be a reason why Girschik’s SVMs [49], constructed using the same data, also are not as successful as the SVM models of Yap and Chen. Yap and Chen’s estimates for the EV test set were about as accurate as for the training models. Girschik [49] pointed out that many of the outliers in the Yap and Chen dataset were in the training set, and that this increased model performance with respect to EV.

In the present work, the rates of correct classification calculated for the SDAR LDA and SAR DF models using the EV set were greater than respective tenfold CV statistics calculated for the training set. The obtained rates of correct classification for SDAR LDA and SAR DF classifiers in excess of 70% on the EV set were in agreement with EV results of Kriegl *et al*. [50] obtained for a three-class SVM model of CYP3A4 inhibitors.

When the EV statistics calculated for the SDAR LDA and SAR DF models were compared with calculations carried out using the ACD/ADME^™^ suite, the comparison confirmed the trends. Although, the training set of ACD/ADME^™^ models was approximately tenfold greater, the commercial software did not perform better than the presented models. For both CYP3A4 (Table 3) and CYP2D6 (Table 6), the accuracy of ACD/ADME^™^ models was lower than that of SDAR LDA and SAR DF models. Sensitivity and specificity roughly followed the trend. Interestingly, an “efficient inhibition” model of the ACD/ADME^™^ suite did not perform differently, but rather more unbalanced, compared with the “general inhibition” model. A sensitivity-to-specificity ratio of the “efficient inhibition” model was consistently biased down as compared with the “general inhibition” model (e.g., down to 20/97 for CYP3A4). A closer look at the data suggested that the number of true positives and false negatives was reduced in both “efficient inhibition” models, but the classification attribution of the retained compounds did not change. Perhaps a similar effect could be achieved by shifting the probability cutoff of the “general inhibition” model from the default value of 0.5. Thus, shifting a threshold of HTS inhibition data categorization does not likely commit new knowledge to machine learning, but rather amounts to reshuffling the data. The latter can be achieved by a more straightforward statistical means.

That the superior size of the training set of ACD/ADME^™^ model did not result in overwhelming performance improvement points to, perhaps, data quality. It suggests that either the quality of clinical inhibition data is higher (the data are less “noisy”), or that *in vitro* and *in vivo* data are sampled from different processes. More evidence to support both hypotheses can be found in a complementary publication [31].

### 2.4. Application to Environmental Health

The SAR DF and ACD/ADME^™^ models were applied to a diverse set of environmental pollutants that endanger public health at HWS across the United States. When using ACD/ADME^™^, a precautionary approach (*p* > 0.5) suggested that close to 20% of chemicals at HWS may be potential substrates for the CYP3A4 isozyme, and approximately 1% of chemicals at HWS may be potential substrates for CYP2D6, while an inhibitory effect on the isozymes may be expected from 7.5% and 1.5% of chemicals, respectively (data are not shown). A less conservative estimation (*p* > 0.5 and reliability index [*RI*] > 0.4) identified approximately half as many chemicals (close to 10% and 1% of chemicals as substrates for CYP3A4 and CYP2D6, respectively, and approximately 5% and 1% as inhibitors). A list of purported chemicals can be found in the supplementary materials.

A rigorous applicability domain has not been defined for the models reported in the present work; neither has it been defined for the ACD/ADME^™^ software. Clearly, the models described in the present report were developed and validated using FDA-approved medications as a training set, while ACD/ADME^™^ CYP models were trained using, perhaps, drug-like compounds. Therefore, application of both sets of models to environmental pollutants can hardly be argued as perfect. Nevertheless, the obtained results seem to pass several general asymptotic tests. First, substrate-indiscriminant CYP3A4 is known to be responsible for metabolism of almost 50% of xenobiotics [3], whereas a more substrate-selective CYP2D6 enjoys a lesser share. The aforementioned estimates meet this condition. Second, while metabolism of a wide range of xenobiotics is the main function of studied CYPs, not all xenobiotics are CYP inhibitors. Therefore, it can be expected that the number of inhibitors in the environment shall not exceed the number of substrates. The reported numbers meet this expectation.

How much the HWS chemicals are representative of chemicals in trade and manufacturing remains to be seen. Perhaps, they represent 2%–5% of the total amount [51]. Caution is advised when generalizing from the HSW numbers to ambient environment.

## 3. Experimental

### 3.1. Data Selection

Query of the PubChem^™^ database [40] for CYP3A4 and CYP2D6 yielded 1,333 and 831 bioassays, respectively; but only two of them for each isozyme were the HTS bioassays in which more than 100 inhibitors have been tested. These were bioassays with assay identification numbers (AIDs) 884, 891, and 1851—all deposited by the National Institutes of Health Chemical Genomics Center. AID 1851 was a multi-assay that contained information for both CYP3A4 and CYP2D6 and three other CYP isoforms. A tested outcome in this bioassay was “whether a compound inhibited pro-luciferin conversion with *any* of the five isozymes” [52], which made delineation of CYP3A4 and CYP2D6 inhibition activities impossible. However, the bioassay also contained a type-identity tag “approved drug” (the other two bioassays did not). There were 1,114 drugs among the 16,555 compounds tested in this bioassay, which represents 6.7% coverage. Despite similarly large sizes of the other two bioassays, comprising more than 13,000 and 9,000 compounds; based on this percentage, coverage of the chemical space available in PubChem^™^ by drugs appeared to be low, *i.e.*, these bioassays were under-representative of the chemical space of actual drugs.

The Yap and Chen [41] compilation of drugs was considered as an alternative. Of these data, 602 compounds were used as a training set for supervised learning in the SDAR and SAR model development. In this training set, there were 216 inhibitors and 386 non-inhibitors of CYP3A4, and 160 inhibitors and 442 non-inhibitors of CYP2D6. An additional set of 100 compounds was withheld from the modeling efforts on 602 compounds and was used for EV of the models.

Of 702 compounds used in the present work, 260 overlapped with the PubChem^™^ bioassay AID 884 for CYP3A4, of which 69 were bioassay inconclusive; 172 overlapped with AID #891 for CYP2D6, including 17 inconclusive; and 165 overlapped with AID 1851 for both CYP3A4 and CYP2D6. In other words, less than 25% of the drugs used in the present work (27%, 22%, and 24%, respectively) were represented in PubChem^™^, which confirms aforementioned poor coverage of FDA-approved drugs in the PubChem^™^ data. Based on these findings and aforementioned considerations, the curated compilation of clinical drugs [41] was chosen for both machine learning and model validation.

Information about environmental health perpetrators was extracted from the Agency for Toxic Substances and Disease Registry (ATSDR) EH Portfolio^™^ (former ATSDR HazDat^™^ database) [53]. EH Portfolio^™^ tracks information about chemical pollutants found at HWS of the National Priority List. After removing mixtures, inorganic compounds, and structural duplicates, 2,170 pure organic compounds (of 3,363 hazardous agents found in the data records of EH Portfolio^™^) were retained for the analysis. The collection was diverse, with chemical molecular weights ranging from 16 Da for methane to 959 Da for decabromodiphenyl ether. Representative chemical classes included dioxins, polychlorinated biphenyls, persistent organic pollutants, organophosphates of all kinds, and industrial solvents.

### 3.2. SDAR Models

**Figure 1:**
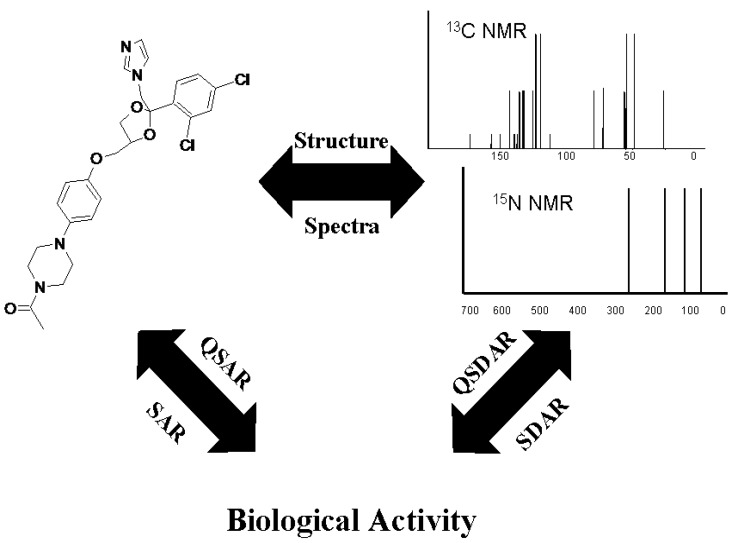
Triangular relationship between the structure, ^13^C- and ^15^N-NMR spectral data, and biological activity. Kectoconazole is used as an example.

SDAR modeling relates the quantum mechanical information at each carbon or nitrogen nucleus to the modeled biological activity of a chemical compound. NMR chemical shifts can be sensitive to the electronic orbitals around the nucleus, delocalization of the electronic charge, and changes in the three-dimensional electrostatic potential around the nucleus. The use of chemical shifts as machine-learning structural descriptors represents a hybrid between a typical SAR approach that relates sub-structural fragments of a compound to its activity and a 3D-QSAR approach that relates physical fields around a chemical to its activity [21]. Figure 1 demonstrates the triangular relationship between the structure of a molecule, chemical NMR spectra, and biological activity. The relationship is true for any molecule. SAR and QSAR take direct advantage of the structure-activity leg of the triangle. SDAR mimics the same relationship indirectly, taking advantage of the other two legs. The indirect approach confers advantages in simplicity of implementation and objectivity of model building. In most but not all cases, it has also shown improved accuracy and estimation performance compared with SAR and QSAR [21,22,23,24].

For each chemical, ^13^C- and ^15^N-NMR spectra were estimated by using, respectively, the ACD/CNMR Predictor^™^ and ACD/NNMR Predictor^™^ modules of ACD/Labs^™^ software, version 12.0, (Advanced Chemistry Development, Inc., Toronto, ON, Canada); the estimated chemical shifts were exported into a text file. The ^13^C-NMR spectrum of each compound in the dataset was binned using custom-built, in-house software with bin widths of 1 ppm (C1), 2 ppm (C2), and 3 ppm (C3). Using a similar approach, each ^15^N-NMR spectrum was binned with widths of 5 ppm (N5), 10 ppm (N10), and 15 ppm (N15).

There were two reasons as to why the chemical-shift range covered by ^15^N bins was wider than the range covered by ^13^C bins; one reason was physical and another was statistical. The physical reasoning suggested that the gyromagnetic ratio of ^13^C is approximately 0.25 of a proton, while the absolute gyromagnetic ratio for ^15^N is approximately 0.10 of a proton. The difference in gyromagnetic properties of the nuclei translated into a wide spectral range for ^15^N (of approximately 600 to 700 ppm) as compared with the range for ^13^C (of about 240 ppm). On the statistical side, there were less nitrogen than carbon atoms in the analyzed compounds. Therefore, the ^15^N bins had to be wider to populate the bins to a similar statistically-significant extent as the by ^13^C bins.

All statistical multiple linear discriminant analyses were performed using Statistica^®^ version 8.0 software from StatSoft, Inc. (Tulsa, OK, USA). LDA training models were built for three sets of ^13^C and ^15^N combination bin widths. The SDAR LDA training classification of inhibitors and non-inhibitors was carried out using the *F*-value forward stepwise analysis criterion, by which a bin was either included in or excluded from the model. Two criteria, either *F* > 0.5 or *F* > 2.0, were applied. All SDAR LDA models were constructed with prior probabilities of classifying a compound as an inhibitor or non-inhibitor set as equal [54]. Setting the prior probability to equal as opposed to a percentage, aided in overcoming the unequal distribution of the two classes being modeled [54].

### 3.3. SAR Methods

Molecular descriptors are intended to extract the structural information in the form of a numerical or graph-digital representation that is suitable for model development. They serve as the bridge between the molecular structure and biological activity of a chemical. Mold^2^ was developed at the U.S. FDA National Center for Toxicological Research as a software package for calculating 777 molecular descriptors [48]. Using Mold^2^, the descriptors for 699 of the 702 compounds were derived solely from their 2D chemical structures (excluding three intricate compounds, cyclosporin, everolimus, and valpodar). Shannon entropy, also called the information entropy, was used to filter out descriptors with low information content. This resulted in 353 molecular descriptors, which were used in the SAR DF model development. DF, a classification method developed in the laboratories of W.T. and H.H. [55,56,57], is a novel pattern-recognition method that combines the results of multiple distinct but comparable decision tree models to reach consensus estimation. At the training stage, Gini's diversity index was used to split the nodes in the decision trees. A consensus among five decision trees was used to construct the final classification models of inhibitors of CYP3A4 and CYP2D6 isozymes. Six to 17 descriptors per tree were used in the final SAR DF models. The resulting most informative descriptors with the assignments of compounds as inhibitor/non-inhibitor of CYP3A4 and CYP2D6 isozymes were used to construct DDCI classifiers.

### 3.4. Cross-Validation and External Validation of SAR and SDAR Models

Tenfold CV was used to evaluate the classification performances of SAR DF and SDAR LDA models. For each tenfold CV, the training set was first randomly divided into ten equal portions; next, each portion was successively excluded from the training set and then estimated by the model developed from the remaining nine portions. The classification statistics of CV were averaged over the ten generated models. Although the division of the dataset was random, still the classification statistics could be biased. To increase the objectivity of analysis, tenfold CV was repeated 100 times. The averaging was expected to decrease the statistical bias of the rate of correct classification, sensitivity, and specificity of the models.

As additional rigor to validation and testing, SAR and SDAR models were also challenged with an EV set of 100 chemicals. This set was one of six EV sets generated by Yap and Chen [41]. The authors have also described a procedure for recruiting compounds in the EV set. EV sets generated by Yap and Chen were internally diverse, as measured by the diversity index (0.002 < DI < 0.020), and approximately equidistant to the reciprocal training set, as given by the representativeness index (0.446 < RI < 0.511) [41]. The latter suggests that the EV set used in the present work was within the chemical space of the training set, and that the EV set is suitable for assessing the models developed within the chemical space of the training set. The training, CV, and EV results were measured in terms of sensitivity, specificity, and the rate of correct classification.

The EV statistics were compared with those obtained using an ACD/ADME^™^ software suite version 5.0 from Advanced Chemistry Development, Inc. A CYP inhibition module of the software has been developed using large libraries of HTS data [58], alike another publication from ACD/Labs^™^ [59]. In that publication, the authors describe a machine-learning classifier trained by using CYP3A4 inhibition data from the AID 884 bioassay of PubChem^™^ [40]. AID 884 reports a percentage of substrate inhibition as the source of data interpretation, from which the IC_50_ is derived. IC_50_ can be modeled either quantitatively [27] or categorically [58,59]. For the latter, software developers have to pick an arbitrary categorization threshold, because the IC_50_ value itself does not substitute for the potency of *in vivo* inhibition, although it could be extrapolated [31]. Two thresholds, 50 μM and 10 μM, have been used by ACD/Labs^™^ to transform IC_50_ data to a form suitable for classifier development. The 50 μM classifier has been called a “general inhibition” model, and the 10 μM classifier has been called an “efficient inhibition” model [58]. With both models, classification was carried out using a probability cutoff of 0.5 and *RI* cutoff of 0.4, per instructions from the ACD/Labs^™^ technical support. The ACD/ADME^™^ inhibition models were applied to the EV set of the present work and the results were compared. Assessment of environmental chemicals involved both CYP-inhibition and CYP-substrate modules of the ACD/ADME^™^ suite. Substrates were selected using the same probability and *RI* cutoffs as inhibitors.

## 4. Conclusions

The present work demonstrated that by using a SDAR LDA and/or SAR DF approach, a supervised machine-learning classifier of a fair accuracy, sensitivity, and specificity can be built for classification of inhibitors and non-inhibitors of major liver CYP isozymes. The rates of correct classification, sensitivity, and specificity of the SDAR LDA and SAR DF models were greater during the EV than during tenfold CV; this circumstance may be due either to statistical power (CV statistics were averaged on a volume of data 600 times larger than the EV ones), or, more likely, to the possibility that the EV set of the Yap and Chen [41], borrowed for the present study, has been over-fitted to the chemical space of the training set [49]. It is also possible that some of the inhibitors in the training and EV sets are only inhibitors for a specific genetic polymorphism of CYP3A4 or CYP2D6—for instance, it has been demonstrated that some drugs can be metabolized differently by certain genetic polymorphisms of CYP3A4 [60] and CYP2D6 [61,62]. Both environmental and genetic components play a role in drug metabolism [63], and until both arrays of information are collected in clinical studies, modeling them accurately would be impossible.

The described machine-learning classifiers were developed without taking into account stereochemical [62] and regiochemical [64] selectivity of CYP-mediated oxidation. Regioselectivity and localization of the site of metabolism (SOM) on the substrate is an important and complementary topic to modeling CYP inhibition. Computational determination of SOMs receives an increasing attention in the literature in recent years [65,66,67,68]. The SOM localization approaches include quantum chemical calculations, molecular docking, molecular shape alignment, molecular field analyses, QSAR, probabilistic and rule-based schemes. Because of the diversity and complexity of these approaches the regioselectivity considerations were not explicitly included in the present study. In the future, they may provide an additional dimension to improving the CYP inhibition modeling.

Regulation of CYP enzymes is a complex phenomenon that involves competitive, non-competitive, uncompetitive, mixed, and mechanism-based inhibition, along with product inhibition, inhibition at the transporter level, and other mechanisms. For example, CYP enzymes can transform chemicals, such as troleandomycin, diltiazem, and tamoxifen, into reactive intermediates that cause mechanism-based inhibition by forming a covalent complex with the heme of the CYP3A4 isozyme [69], while other inhibitors are reversible and interact with CYP in either a competitive or non-competitive manner [70], and can be studied by molecular docking [31,71]. In principle, embedding multiple chemical and genetic mechanisms implicitly in the same machine-learning classifier model is possible, provided each of them is represented by a sufficient number of compounds in the training set. At this point we have no information either about how many mechanisms of inhibition are represented in the training set or about the power of each mechanisms-of-inhibition category, which certainly may affect the model accuracy. Also, because of the diverse mechanisms, initial misattribution of the data utilized as a training set is possible. Confounding information in the training set is never helpful. For instance, Yamashita *et al*. [72] has developed and applied an artificial intelligence system to extract the information concerning interactions of drugs and chemicals with CYP enzymes. Many compounds used in the present study were categorized differently by Yamashita [72], which attests to the difficult nature of modeling the CYP inhibition. Thus, a high level of modeling detail is unfeasible at present, because the exact mechanisms of action of many inhibitors are not yet known, as well as the involved genetic components.

The SDAR LDA models for CYP3A4 and CYP2D6 had very little overlap in the bins used to develop their respective models. This type of information may be useful in understanding and estimating chemical inhibition of CYP enzymes. A clear trend in the SDAR LDA model classification accuracy with respect to bin sizes was observed. Further evaluation of the effect of ^13^C and ^15^N bin sizes is needed for fine-tuning the SDAR models in the future. Another way to improve model quality may be 3D-SDAR modeling, which uses spectral NMR information with atom-to-atom structural information before relating this combined information to biological activity [73,74,75]. It is expected that the power of inhibitor/non-inhibitor classification will be greater using 3D-SDAR than 1D-SDAR presented here.

The environmental health significance of models parameterized on pharmaceuticals remains to be seen. Preliminary screening of HWS chemicals suggests that an appreciable number of them may interfere with the CYP biotransformation system. Because the levels of exposure to environmental pollutants are typically not high, the physiologic inhibitory effect of pollutants on medicated patients may be insignificant, but not the other way round. The ability to excrete toxic environmental chemicals may be significantly impaired in patients medicated with potent CYP inhibitors and, thus, the toxic response to environmental chemicals amplified. Similar considerations apply to nutritional supplements and dietary sources. For instance bergamottin, a natural ingredient of grapefruit juice, is a potent irreversible inhibitor of CYP3A4 that affects the first-pass metabolism [76,77,78,79]. As a result, clinical interactions of grapefruit juice with drugs, especially diazepam, midazolam, triazolam, and other benzodiazepines, are significant [80,81]. Similarly resveratrol, a trendy anti-ageing supplement with anti-angiogenic, anti-inflammatory, anti-diabetic, anti-adipogenic and neuroprotective action, that may be effective at high doses of as much as 1,000 mg/kg/day [82,83], is also a mechanism-based inhibitor of CYP3A4 [79]. Thorough examination of large chemical libraries, such as the Natural Products subset of the ZINC database [84], using computational screening methods, similar to those described in the present report, may help to reduce uncertainties related to co-administration of drugs, environmental pollutants, and natural products. Proactive knowledge of such hidden drug-chemical interaction may help improve the environmental health assessment in communities and near hazardous waste sites.

## Supplementary Materials

Supplementary materials can be accessed at: http://www.mdpi.com/1420-3049/17/3/3383/s1.
