# Supplementary material for: Modeling Chemical Interaction Profiles: I. Spectral Data-Activity Relationship and Structure-Activity Relationship Models for Inhibitors and Non-inhibitors of Cytochrome P450 CYP3A4 and CYP2D6 Isozymes
Source: Molecules. 2012 Mar 15;17(3):3383–406. doi: 10.3390/molecules17033383 (PMC6268752; doi:10.3390/molecules17033383)
Supplement: Supplementary file 1 [file molecules-17-03383-s001.pdf]

# Supplementary Material

# Modeling Chemical Interaction Profiles: I. Spectral Data-Activity Relationship and Structure-Activity Relationship Models for Inhibitors and Non-inhibitors of Cytochrome P450 CYP3A4 and CYP2D6 Isozymes

**Table 1.** Tentative substrates and inhibitors of CYP3A4 isozyme. The calculations were carried out for hazardous waste chemicals recorded in the ATSDR EH Portfolio using the P450 substrate and P450 inhibitor modules of the ACD/ADME suite from Advanced Chemistry Development, Inc. (Toronto, ON, Canada). A probability (*p*) cutoff of 0.5 and reliability index (*RI*) cutoff of 0.4 were applied. Several compounds, whose activity was confirmed by screening the literature, are shown in **bold** (but an extensive systematic search for all compounds was not performed at this time).

| | **3A4** | |
|---|---|---|
| **CAS** | **Substrates** | **Inhibitors** |
| 3218-36-8 | (1,1a-biphenyl)-4-carboxaldehyde | |
| 39001-02-0 | 1,2,3,4,6,7,8,9-octachlorodibenzofuran | |
| 67562-39-4 | 1,2,3,4,6,7,8-heptachlorodibenzofuran | |
| 35822-46-9 | 1,2,3,4,6,7,8-heptachlorodibenzo-p-dioxin | |
| 70648-25-8 | 1,2,3,4,6,7,9-heptachlorodibenzofuran | |
| 58200-70-7 | 1,2,3,4,6,7,9-heptachlorodibenzo-p-dioxin | |
| 69698-58-4 | 1,2,3,4,6,8,9-heptachlorodibenzofuran | |
| 55673-89-7 | 1,2,3,4,7,8,9-heptachlorodibenzofuran | |
| 38178-99-3 | 1,2,4,5,7,8-hexachoro-(9H)-xanthene | |
| 470-82-6 | 1,8-cineole | |
| 28548-08-5 | 12-ketoendrin | 12-ketoendrin |
| 132861-79-1 | 15-tetracosynoic acid, methyl ester | 15-tetracosynoic acid, methyl ester |
| 2597-11-7 | | 1-hydroxychlordene |
| 832-69-9 | 1-methylphenanthrene | |
| 2381-21-7 | 1-methylpyrene | |
| 5566-34-7 | Gamma-chlordane | Gamma-chlordane |
| 2051-24-3 | decachlorobiphenyl | |
| 40186-72-9 | 2,2´,3,3´,4,4´,5,5´,6-nonachlorobiphenyl | |
| 35694-08-7 | 2,2´,3,3´,4,4´,5,5´-octachlorobiphenyl | |
| 52663-79-3 | 2,2´,3,3´,4,4´,5,6,6´-nonachlorobiphenyl | |
| 42740-50-1 | 2,2´,3,3´,4,4´,5,6´-octachlorobiphenyl | |
| 35065-30-6 | 2,2´,3,3´,4,4´,5-heptachlorobiphenyl | |
| 33091-17-7 | 2,2´,3,3´,4,4´,6,6´-octachlorobiphenyl | |
| 52663-71-5 | 2,2´,3,3´,4,4´,6-heptachlorobiphenyl | |
| 52663-77-1 | 2,2´,3,3´,4,5,5´,6,6´-nonachlorobiphenyl | |
| 52663-75-9 | 2,2´,3,3´,4,5,5´,6´-octachlorobiphenyl | |
| 68194-17-2 | 2,2´,3,3´,4,5,5a6-octachlorobiphenyl | |
| 52663-74-8 | 2,2´,3,3´,4,5,5´-heptachlorobiphenyl | |

**Table 1.** *Cont.*

| | 3A4 | |
|---|---|---|
| **CAS** | **Substrates** | **Inhibitors** |
| 52663-73-7 | 2,2′,3,3′,4,5,6,6′-octachlorobiphenyl | |
| 38411-25-5 | 2,2′,3,3′,4,5,6′-heptachlorobiphenyl | |
| 68194-16-1 | 2,2′,3,3′,4,5,6-heptachlorobiphenyl | |
| 40186-71-8 | 2,2′,3,3′,4,5′,6,6′-octachlorobiphenyl | |
| 52663-70-4 | 2,2′,3,3′,4,5′,6′-heptachlorobiphenyl | |
| 40186-70-7 | 2,2′,3,3′,4,5′,6-heptachlorobiphenyl | |
| 55215-18-4 | 2,2′,3,3′,4,5-hexachlorobiphenyl | |
| 52663-65-7 | 2,2′,3,3′,4,6,6′-heptachlorobiphenyl | |
| 61798-70-7 | 2,2′,3,3′,4,6-hexachlorobiphenyl | |
| 2136-99-4 | 2,2′,3,3′,5,5′,6,6′-octachlorobiphenyl | |
| 52663-67-9 | 2,2′,3,3′,5,5′,6-heptachlorobiphenyl | |
| 52663-64-6 | 2,2′,3,3′,5,6,6′-heptachlorobiphenyl | |
| 52704-70-8 | 2,2′,3,3′,5,6-hexachlorobiphenyl | |
| 38411-22-2 | 2,2′,3,3′,6,6′-hexachlorobiphenyl | |
| 52663-76-0 | 2,2′,3,4,4′,5,5′,6-octachlorobiphenyl | |
| 35065-29-3 | 2,2′,3,4,4′,5,5′-heptachlorobiphenyl | |
| 74472-52-9 | 2,2′,3,4,4′,5,6,6′-octachlorobiphenyl | |
| 60145-23-5 | 2,2′,3,4,4′,5,6′-heptachlorobiphenyl | |
| 74472-47-2 | 2,2′,3,4,4′,5,6-heptachlorobiphenyl | |
| 52663-69-1 | 2,2′,3,4,4′,5′,6-heptachlorobiphenyl | |
| 74472-48-3 | 2,2′,3,4,4′,6,6′-heptachlorobiphenyl | |
| 52712-05-7 | 2,2′,3,4,5,5′,6-heptachlorobiphenyl | |
| 74472-49-4 | 2,2′,3,4,5,6,6′-heptachlorobiphenyl | |
| 68194-15-0 | 2,2′,3,4,5,6′-hexachlorobiphenyl | |
| 68194-14-9 | 2,2′,3,4,5′,6-hexachlorobiphenyl | |
| 52663-68-0 | 2,2′,3,4′,5,5′,6-heptachlorobiphenyl | |
| 74487-85-7 | 2,2′,3,4′,5,6,6′-heptachlorobiphenyl | |
| 68194-08-1 | 2,2′,3,4′,6,6′-hexachlorobiphenyl | |
| 68194-09-2 | 2,2′,3,5′,6,6′-hexachlorobiphenyl | |
| 33979-03-2 | 2,2′,4,4′,6,6′-hexachlorobiphenyl | |
| 74472-53-0 | 2,3,3′,4,4′,5,5′,6-octachlorobiphenyl | |
| 39635-31-9 | 2,3,3′,4,4′,5,5′-heptachlorobiphenyl | |
| 41411-64-7 | 2,3,3′,4,4′,5,6-heptachlorobiphenyl | |
| 74472-50-7 | 2,3,3′,4,4′,5′,6-heptachlorobiphenyl | |
| 74472-51-8 | 2,3,3′,4,5,5′,6-heptachlorobiphenyl | |
| 39635-35-3 | 2,3,3′,4,5,5′-hexachlorobiphenyl | |
| 74472-43-8 | 2,3,3′,4,5′,6-hexachlorobiphenyl | |
| 74472-46-1 | 2,3,3′,5,5′,6-hexachlorobiphenyl | |
| 129-79-3 | | 2,4,7-trinitrofluorenone |
| 3674-66-6 | 2,5-dimethyl-phenanthrene | |
| 54964-82-8 | 2,6,10,14-tetramethyloctadecane | |
| 31551-45-8 | | 2,7-dinitrofluorenone |
| 117-79-3 | | 2-aminoanthraquinone |
| 2698-41-1 | | 2-chlorobenzylidene malononitrile |
| 31081-17-1 | 2-methyl-5-propylnonane | |

**Table 1.** *Cont.*

| 3A4 | | |
|---|---|---|
| **CAS** | **Substrates** | **Inhibitors** |
| 1560-97-0 | 2-methyldodecane | |
| 2531-84-2 | 2-methyl-phenanthrene | |
| 91-94-1 | | 3,3´-dichlorobenzidine |
| 119-90-4 | | 3,3´-dimethoxybenzidine |
| 33954-06-2 | 3-5-dimethylphenanthrene | |
| 56-49-5 | 3-methylcholanthrene | |
| 17312-57-1 | 3-methyldodecane | |
| 101-14-4 | | 4,4´-methylenebis(2-chloroaniline) |
| 92-67-1 | | 4-aminobiphenyl |
| 832-64-4 | 4-methylphenanthrene | |
| 4736-56-5 | 5alpha-stigmast-22-en-3beta-ol | |
| 3697-24-3 | 5-methylchrysene | |
| 57-97-6 | 7,12-dimethylbenz(a)anthracene | |
| 20302-14-1 | | 9,9-diphenylfluorene |
| 779-02-2 | 9-methylanthracene | |
| 34256-82-1 | Acetochlor | |
| 1757-18-2 | Akton | |
| 15972-60-8 | Alachlor | |
| 309-00-2 | Aldrin | Aldrin |
| 56534-02-2 | | Alpha-chlordene |
| 834-12-8 | Ametryn | |
| 92-87-5 | | Benzidine |
| 30777-19-6 | Benzo(b)fluorene | |
| 61089-87-0 | Benzofluorene | |
| 205-39-0 | Benzonaphthofuran | |
| 53-89-4 | | Benzpiperylon |
| 56534-03-3 | | Beta-chlordene |
| 33399-00-7 | Bromfenvinphos | |
| 128-37-0 | | Butylated hydroxytoluene |
| 133-06-2 | | Captan |
| 786-19-6 | Carbophenothion | Carbophenothion |
| 143-50-0 | Chlordecone | Chlordecone |
| 3734-48-3 | | Chlordene |
| 470-90-6 | Chlorfenvinphos | |
| 24934-91-6 | Chlormephos | |
| 26264-07-3 | | Chloroanthraquinone |
| 2921-88-2 | **Chlorpyrifos** | |
| 57-88-5 | Cholest-5-en-3beta-ol | |
| 14982-53-7 | Cholestane | |
| 80-99-9 | Cholestene-3-ol | |
| 218-01-9 | Chrysene | |
| 5103-71-9 | Cis-chlordane | Cis-chlordane |
| 360-68-9 | Coprosterol | |
| 56-72-4 | | Coumaphos |
| 26444-49-5 | | Cresyl diphenyl phosphate |

**Table 1.** *Cont.*

| | 3A4 | |
|---|---|---|
| **CAS** | **Substrates** | **Inhibitors** |
| 7700-17-6 | Crotoxyphos | Crotoxyphos |
| 21725-46-2 | Cyanazine | |
| 2636-26-2 | Cyanophos | Cyanophos |
| 68359-37-5 | | Cyfluthrin |
| 68085-85-8 | | Cyhalothrin |
| 52315-07-8 | | Cypermethrin |
| 950-10-7 | Cytrolane | |
| 789-02-6 | DDT, O,P´- | |
| 50-29-3 | DDT, P,P´- | |
| 52918-63-5 | | Deltamethrin |
| 13684-56-5 | | Desmedipham |
| 333-41-5 | Diazinon | Diazinon |
| 1770-80-5 | Dibutyl chlorendate | Dibutyl chlorendate |
| 2528-36-1 | Dibutyl phenyl phosphate | |
| 115-32-2 | Dicofol | |
| 60-57-1 | Dieldrin | Dieldrin |
| 60-51-5 | Dimethoate | |
| 29062-98-4 | Dimethyl phenanthrene | |
| 78-34-2 | Dioxathion | |
| 298-04-4 | **Disulfoton** | |
| 13560-92-4 | Dechlorane 603 | Dechlorane 603 |
| 68937-40-6 | | Tris(isobutylphenyl) phosphate |
| 68937-41-7 | | Isopropylphenyl phosphate |
| 115-29-7 | Endosulfan | Endosulfan |
| 959-98-8 | Alpha-endosulfan | Alpha-endosulfan |
| 33213-65-9 | Beta-endosulfan | Beta-endosulfan |
| 1031-07-8 | Endosulfan sulfate | Endosulfan sulfate |
| 72-20-8 | Endrin | Endrin |
| 7421-93-4 | Endrin aldehyde | Endrin aldehyde |
| 53494-70-5 | Endrin ketone | Endrin ketone |
| 2104-64-5 | | EPN |
| 4651-51-8 | (3beta)-ergost-5-en-3-ol | |
| 563-12-2 | Ethion | Ethion |
| 13194-48-4 | Ethoprop | |
| 91-53-2 | | Ethoxyquin |
| 22224-92-6 | Fenamiphos | |
| 122-14-5 | Fenitrothion | Fenitrothion |
| 64257-84-7 | | Fenpropathrin |
| 115-90-2 | Fensulfothion | Fensulfothion |
| 55-38-9 | Fenthion | Fenthion |
| 69770-45-2 | | Flumethrin |
| 85509-19-9 | | Flusilazole |
| 944-22-9 | Fonophos | Fonophos |
| 56641-38-4 | | Gamma-chlordene |
| 86-50-0 | | Guthion |

**Table 1.** *Cont.*

| | 3A4 | |
|---|---|---|
| **CAS** | **Substrates** | **Inhibitors** |
| 76-44-8 | Heptachlor | Heptachlor |
| 1024-57-3 | Heptachlor epoxide | Heptachlor epoxide |
| 2440-02-0 | | Heptachloronorbornene |
| 70-30-4 | Hexachlorophene | Hexachlorophene |
| 16958-85-3 | Hexadecanoic acid, octyl ester | |
| 630-06-8 | Hexatriacontane | |
| 2899-02-7 | 1,3-dichloro-1,3-bis(2,4,6-trichlorophenyl)urea | |
| 42509-80-8 | Isazophos | |
| 465-73-6 | Isodrin | Isodrin |
| 1617-70-5 | Lupenone | |
| 121-75-5 | Malathion | Malathion |
| 57837-19-1 | Metalaxyl | |
| 301-12-2 | Metasystox R | |
| 950-37-8 | Methidathion | |
| 2032-65-7 | Methiocarb | |
| 41637-90-5 | Methylchrysene | |
| 29253-36-9 | Methylethylnaphthalene | |
| 298-00-0 | Methyl parathion | Methyl parathion |
| 953-17-3 | Methyl trithion | Methyl trithion |
| 51218-45-2 | Metolachlor | |
| 2385-85-5 | Mirex | Mirex |
| 5103-73-1 | Cis-nonachlor | Cis-nonachlor |
| 39765-80-5 | Trans-nonachlor | Trans-nonachlor |
| 18181-70-9 | Iodofenphos | Iodofenphos |
| 2524-09-6 | o,o,s-triethyl-dithiophosphate | |
| 32536-52-0 | Octabromodiphenyl-ether | |
| 31472-83-0 | Octachlorobiphenyl | |
| 706-78-5 | | Octachlorocyclopentene |
| 3268-87-9 | Octachlorodibenzo-*p*-dioxin | |
| 112516-18-4 | Octahydrotrimethyl-(1-methylethyl)-phenanthrene | Octahydrotrimethyl-(1-methylethyl)-phenanthrene |
| 511-15-9 | Octahydrotrimethyl(methylethyl)-phenanthrenol | |
| 27304-13-8 | Oxychlordane | Oxychlordane |
| 56-38-2 | Parathion | Parathion |
| 630-07-9 | Pentatriacontane | |
| 86508-42-1 | Perfluoro compounds, c5–18 | |
| 77-09-8 | | Phenolphthalein |
| 298-02-2 | **Phorate** | |
| 13366-73-9 | | Photodieldrin |
| 39801-14-4 | Photomirex | Photomirex |
| 150-86-7 | Phytol | |
| 51-03-6 | | **Piperonyl butoxide** |
| 311-45-5 | **Paraoxon** | **Paraoxon** |

**Table 1.** *Cont.*

| | 3A4 | |
|---|---|---|
| **CAS** | **Substrates** | **Inhibitors** |
| 1610-18-0 | Prometon | |
| 7287-19-6 | Prometryn | |
| 2312-35-8 | Propargite | |
| 139-40-2 | Propazine | |
| 34643-46-4 | Prothiophos | |
| 299-84-3 | Ronnel | |
| 83-79-4 | | Rotenone |
| 78-48-8 | **Tribufos** | |
| 94-59-7 | | Safrole |
| 85-60-9 | 4,4´-butylidenebis(6-tert-butyl-3-methylphenol) | |
| 515-03-7 | Sclareol | Sclareol |
| 1014-70-6 | Simetryn | |
| 601-58-1 | Stigmastane | |
| 8001-50-1 | Strobane | Strobane |
| 57-24-9 | Strychnine | |
| 3689-24-5 | Sulfotep | |
| 35400-43-2 | **Sulprofos** | Sulprofos |
| 88671-89-0 | Systhane | Systhane |
| 1401-55-4 | | Tannin |
| 514-07-8 | Taraxerone | |
| 56803-37-3 | | t-butylphenyl diphenyl phosphate |
| 79538-32-2 | Telfluthrin | |
| 3383-96-8 | Temephos | Temephos |
| 13071-79-9 | Terbufos | |
| 33693-04-8 | Terbumeton | |
| 886-50-0 | Terbutryn | |
| 4181-95-7 | Tetracontane | |
| 116-29-0 | Tetradifon | |
| 107-49-3 | Tetraethyl pyrophosphate | |
| 3590-84-9 | | Tetraoctylstannane |
| 8001-35-2 | Toxaphene | Toxaphene |
| 5103-74-2 | Trans-chlordane | Trans-chlordane |
| 2303-17-5 | Triallate | |
| 126-73-8 | Tributylphosphate | |
| 688-73-3 | Tributyltin | |
| 1330-78-5 | | Tricresyl phosphate |
| 78-30-8 | | Tri-o-cresyl phosphate |
| 869-59-0 | | Trioctylstannane |
| 603-34-9 | Triphenyl amine | |
| 217-59-4 | Triphenylene | |
| 639-58-7 | Triphenyltin chloride | |
| 126-72-7 | Tris(2,3-dibromopropyl) phosphate | |
| 6145-73-9 | Tris(2-chloropropyl) phosphate | |
| 25155-23-1 | | Trixylyl phosphate |
| 81-81-2 | Warfarin | |

**Table 2.** Tentative substrates and inhibitors of CYP2D6 isozyme. The calculations were carried out for hazardous waste chemicals recorded in the ATSDR EH Portfolio using the P450 substrate and P450 inhibitor modules of the ACD/ADME suite from the Advance Chemistry Development, Inc. (Toronto, ON). A probability (*p*) cutoff of 0.5 and reliability index (*RI*) cutoff of 0.4 were applied. Several compounds, whose activity was confirmed by screening the literature, are shown in **bold** (but an extensive systematic search for all compounds was not performed at this time).

| | **2D6** | |
|---|---|---|
| **CAS** | **Substrates** | **Inhibitors** |
| 538-74-9 | | Dibenzyl sulphide |
| 1165-57-7 | | 1-phenyl-2-(3-phenylphenyl)benzene |
| 95-73-8 | | 2,4-dichlorotoluene |
| 612-94-2 | | 2-phenylnaphthalene |
| 1165-14-6 | | 1,2,3-tri(phenyl)benzene |
| 203-64-5 | | 4H-cyclopenta(def)phenanthrene |
| 486-60-2 | | 5-hydroxy-6,7-furanocoumarin |
| 260-94-6 | Acridine | |
| 30777-19-6 | | Benzo(b)fluorene |
| 207-08-9 | | Benzo(k)fluoranthene |
| 61089-87-0 | | Benzofluorene |
| 53-89-4 | | Benzpiperylon |
| 786-19-6 | Carbophenothion | |
| 298-04-4 | **Disulfoton** | |
| 563-12-2 | Ethion | |
| 91-53-2 | | Ethoxyquin |
| 55-38-9 | Fenthion | |
| 944-22-9 | Fonophos | |
| 58-27-5 | | Menadione |
| 537-46-2 | **Methamphetamine** | Methamphetamine |
| 27323-29-1 | Methylcarbazole | |
| 953-17-3 | Methyl trithion | |
| 298-02-2 | **Phorate** | |
| 78-48-8 | S,S,S-tributyl phosphorotrithioate | |
| 35400-43-2 | **Sulprofos** | |
| 13071-79-9 | Terbufos | |
| 869-59-0 | | Trioctylstannane |
